# New Horizons in Chemical Functionalization of Endohedral Metallofullerenes

**DOI:** 10.3390/molecules25163626

**Published:** 2020-08-10

**Authors:** Michio Yamada, Michael T. H. Liu, Shigeru Nagase, Takeshi Akasaka

**Affiliations:** 1 Department of Chemistry, Tokyo Gakugei University, Koganei, Tokyo 184-8501, Japan; 2 Department of Chemistry, University of Prince Edward Island, Charlottetown, PE C1A4P3, Canada; diazirine@gmail.com; 3 Fukui Institute for Fundamental Chemistry, Kyoto University, Sakyo-ku, Kyoto 606-8103, Japan; nagase@ims.ac.jp; 4 TARA Center, University of Tsukuba, Tsukuba, Ibaraki 305-8577, Japan; 5 Foundation for Advancement of International Science, Tsukuba, Ibaraki 305-0821, Japan; 6 State Key Laboratory of Materials Processing and Dye and Mold Technology, School of Materials Science and Engineering, Huazhong University of Science and Technology, Wuhan 430074, China

**Keywords:** addition reactions, carbenes, chemical functionalization, electrochemistry, endohedral metallofullerene, fullerene, lanthanide ions, nanocarbon, pericyclic reactions, radicals

## Abstract

This overview explains some new aspects of chemical functionalization of endohedral metallofullerenes (EMFs) that have been unveiled in recent years. After differences in chemical reactivity between EMFs and the corresponding empty fullerenes are discussed, cage-opening reactions of EMFs are examined. Then, the selective bisfunctionalization of EMFs is explained. Finally, single-bonding derivatization of EMFs is addressed. The diversity and applicability of the chemical functionalization of endohedral metallofullerenes are presented to readers worldwide.

## 1. Introduction

Endohedral metallofullerenes (EMFs) are hybrid molecules of spherical nanocarbons, known as fullerenes, with atomic metal(s) or metal-containing clusters encaged inside. Unique molecular structures of this kind have fascinated many researchers in many fields, including chemistry, physical science, and materials science. Soon after the discovery of C_60_, EMFs were first reported in 1985 by Smalley and coworkers [1]. Findings from early studies of their synthesis, structures, and properties were summarized in several reports [2,3,4,5]. In addition, research progress after 2010 has been described in several published reviews [6,7,8,9,10,11,12,13]. Recent progress on silylation and germylation of EMFs was reviewed by Kako et al. [14] However, several chemical reactions of EMFs have been developed since. Various molecular structures of EMF derivatives have been clarified one after another. This review was undertaken to overview the most recent advances in the chemistry of EMFs developed in the last decade, particularly addressing chemical functionalization.

## 2. Differences in Chemical Reactivity between EMFs and the Corresponding Empty Fullerenes

The chemical reactivities of EMFs differ from those of empty fullerenes, because of their different carbon-cage geometries and their different redox properties. Comparison of the chemical reactivities of EMFs with those of empty fullerenes is expected to provide deeper understanding of how endohedral metal-atom doping affects the chemical reactivity of fullerenes. However, the difficulty of pursuing this subject experimentally is that most carbon cages of EMFs are not available in their pristine forms. Empty fullerenes and EMFs are synthesized, generally by arc-discharging of graphitic carbon containing metal species such as metal oxides. Because of the intense temperature (2000–3000 K) conditions, the resulting fullerene species are regarded not as kinetically but as thermodynamically favorable ones. At this point, the presence or absence of ‘intramolecular’ electron transfer from the inner metal atom(s) to the outer carbon cages drastically alters the thermodynamic stability of the carbon cages. For instance, adjacent pentagonal rings are disfavored in the carbon cages of empty fullerenes. Therefore, empty fullerenes generally satisfy the empirical rule, the so-called isolated pentagon rule (IPR), which states that stable fullerenes have each of their 12 pentagonal rings surrounded by five hexagonal rings [15]. In fact, fusing pentagonal rings increases the local steric strain caused by enforced bond angles, accompanied by the higher pyramidalization of the carbon atoms. In addition, a fused pentagon substructure must be antiaromatic. Nonetheless, the circumstances are different in the case of EMFs [16]. In fact, several EMFs that possess non-IPR carbon cages have been discovered to date [17]. Crystallographic studies of such non-IPR EMFs clearly illustrate that the encaged metal atoms are closely positioned near the fused pentagonal rings on the carbon cages, implying that the encaged metal atoms stabilize the non-IPR carbon cages that are not available in pristine forms without encaged metal atoms. As a result, studies using experimentation to compare the chemical reactivities of empty fullerenes and the EMFs were left unexplored until 2016.

In this respect, we have specifically examined on the *D*_2*d*_(23)-C_84_ carbon cage, because the carbon cage is the sole exception by which both the empty and the endohedral species are thermodynamically stable at ambient conditions. The empty fullerene was first reported in 1992 by Kikuchi, Achiba, and their coworkers [18]. Its isolation was reported by Dennis and Shinohara in 1998 [19]. As indicated in its name, *D*_2*d*_(23)-C_84_ has the molecular symmetry of *D*_2*d*_; it, therefore, consists of the 11 nonequivalent carbon atoms. The number in the parenthesis indicates the spiral code for IPR-satisfying cage structures assigned based on the Fowler–Manolopoulos (FM) spiral algorithm, which is used to designate the cage structure [20]. The corresponding EMF, which contains the same carbon cage, Sc_2_C_2_@*D*_2*d*_(23)-C_84_, was first reported by Shinohara et al. However, it was assigned incorrectly at that time as Sc_2_@C_86_ [21]. The correct structure of the EMF as Sc_2_C_2_@*D*_2*d*_(23)-C_84_ was revised by the same group, based on the maximum entropy method (MEM)/Rietveld analysis of the powder X-ray diffraction data [22]. The revised structure was finally confirmed by Akasaka et al., based on two-dimensional incredible natural abundance double quantum transfer experiment (2D INADEQUATE) NMR studies of its ^13^C-enriched sample [23]. The ‘molecular’ symmetry of Sc_2_C_2_@*D*_2*d*_(23)-C_84_ can be regarded not as *D*_2*d*_, but as *C*_2*v*_. Lowering of the molecular symmetry results from the placement of the internal Sc_2_C_2_ cluster, by which the C_2_ unit is not parallel but rather perpendicular to the main *C*_2_ axis. Nevertheless, the lower availability of both the species hampered further studies of their chemical reactivities.

**Figure 1:**
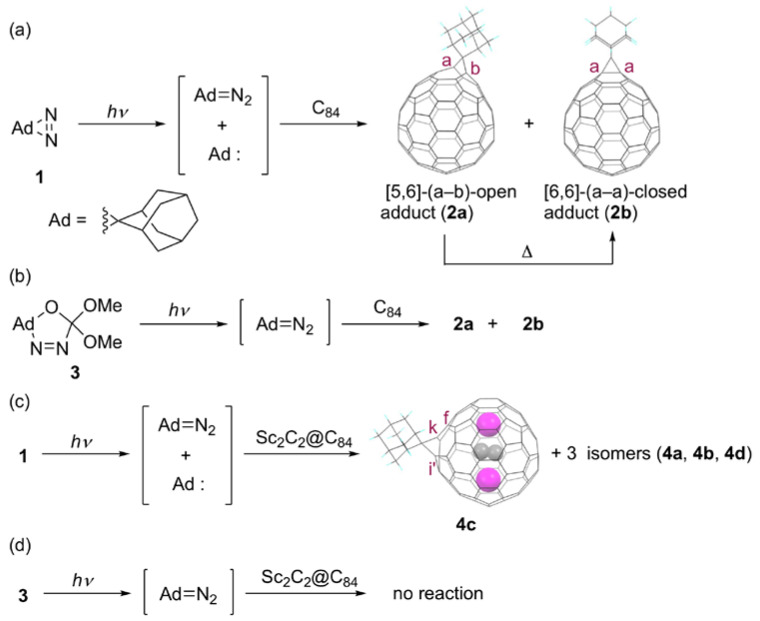
(**a**) Photochemical reaction of C_84_ and **1**. (**b**) Photochemical reaction of C_84_ and **3**. (**c**) Photochemical reaction of Sc_2_C_2_@C_84_ and **1**. (**d**) Photochemical reaction of Sc_2_C_2_@C_84_ and **3**.

We launched a large-scale arc-discharge chamber and optimized both the discharging conditions and the extraction–purification processes, to obtain sufficient amounts of such ‘minor’ fullerene products [8]. Subsequently, we conducted experiments that revealed differences in the chemical reactivity and selectivity between *D*_2*d*_(23)-C_84_ and Sc_2_C_2_@*D*_2*d*_(23)-C_84_ (hereinafter, the molecules will be abbreviated respectively as C_84_ and Sc_2_C_2_@C_84_), for which the photo-reactions of the fullerenes with 2-adamantane-2,3′-[3*H*]-diazirine (**1**) were used for the investigation, as shown in Figure 1 [24]. The photolysis of **1** is well known to generate both adamantylidene carbene Ad: (Ad = adamantylidene) and diazoadamantane as reactive intermediates in a 1:1 ratio, based on laser flash photolysis studies [25]. The photo-reaction of C_84_ and **1** at ambient temperature caused the formation of two mono-adducts, major product **2a** and minor product **2b**, for which the respective conversion yields of **2a** and **2b** based on the reacted C_84_ were 80% and 12% (see Figure 1a). In that case, 52% of C_84_ was consumed during 90 s irradiation using an ultra-high-pressure mercury-arc lamp (cutoff < 350 nm). The **2a** and **2b** structures were assigned respectively to the [5,6]-(a–b)-open fulleroid and the [6,6]-(a–a)-closed methanofullerene, for which the terms [5,6] and [6,6] respectively show that the site of addition occurred at a bond shared by one pentagonal ring and one hexagonal ring, or a bond shared by two hexagonal rings. The structure of **2b** was also ascertained using single-crystal XRD. No interconversion was observed between **2a** and **2b** under photoirradiation. However, **2a** was thermally interconverted to **2b** under heating at 80°C.

To assess the chemical reactivity of C_84_ toward the reactive intermediates, comparative studies of the photolysis of **1** and 5′.5′-dimethoxyspiro[adamantane]-2,2′-[Δ^3^-1,3,4-oxadiazoline] (**3**) in the presence of C_84_ at –78°C were conducted, respectively, because **3** is known to generate diazoadamantane exclusively under photoirradiation (see Figure 1b). On one hand, the photoreaction of C_84_ with **1** caused the formation of **2a** and **2b** in a 10:1 ratio under the condition. On the other hand, the photoreaction of C_84_ with **3** caused the formation of **2a** and **2b** in a ratio of 10:1, which is the same ratio as that obtained in the reaction of C_84_ and **1**. The comparable product ratios imply that in situ generated diazoadamantane contribute mainly to the reaction process in the photolysis of **1** in the presence of C_84_. Combined with density functional theory (DFT) calculations, the scenario of the photoreactions was proposed as follows: first, the regioselective [3 + 2] cycloaddition of C_84_ with in-situ generated diazoadamantane took place at the [6,6]-(a–a) bond, in which the two-fold degenerate LUMO is localized primarily, to give the pyrazoline intermediate. Secondly, thermal decomposition of the pyrazoline intermediate occurred in a concerted pathway, to give **2** exclusively. Finally, a concerted [1,5]-sigmatropic shift of **2a** led to the formation of **2b**.

In contrast to that of the empty fullerene, the photoreaction of Sc_2_C_2_@C_84_ with **1** at ambient temperature yielded four monoadducts, **4a**, **4b**, **4c**, and **4d** in an 8:7:3:1 ratio, respectively, after 1 min (see Figure 1c). Among these, only the structure of **4c** has been elucidated by single-crystal XRD, showing that the third most abundant isomer is the [6,6]-(k–i’)-open fulleroid. No interconversion was observed between **4a** and **4b** under photoirradiation, but they both interconvert to **4c** under thermal conditions. It is noteworthy that no reaction proceeded in the photolysis of **3** in the presence of Sc_2_C_2_@C_84_, indicating that Sc_2_C_2_@C_84_ is not reactive toward diazoadamantane (see Figure 1d). In this context, it is reasonable to consider that the photoreaction of Sc_2_C_2_@C_84_ with **1** proceeded via a carbene addition mechanism, unlike the corresponding empty fullerene. Unfortunately, an experiment on the structural elucidation of **4a**, **4b**, and **4d** has not been conducted. However, based on the hypothesis that the addend must move to bind with an adjacent bond during thermal interconversion and the DFT calculations of the relative energies, **4a** and **4b** can be the [6,6]-(k–k) and [6,6]-(k–f) addition products. Inspection of the molecular orbital (MO) diagrams shows that the LUMO of Sc_2_C_2_@C_84_ is higher in energy than that of C_84_. Therefore, the inertness of Sc_2_C_2_@C_84_ toward diazoadamantane can be derived from the energy mismatch between their frontier orbitals. However, the higher level of the HOMO of Sc_2_C_2_@C_84_ compared to that C_84_ might result in promoting reactivity toward the electrophilic carbene. Nevertheless, the delocalization of the HOMO of Sc_2_C_2_@C_84_ hampers the proper prediction of the reactive sites. The addition sites of the proposed structures of **4a** and **4b**, and the XRD-determined structure of **4c** cannot be explained based either on the *p*-orbital axis vector (POAV) values or on the negative-charge densities. DFT calculations suggest that the formation of **4c** is governed by thermodynamic control. Throughout, their different chemical reactivities of C_84_ and Sc_2_C_2_@C_84_ are derived from Sc_2_C_2_ doping, which raises the HOMO and LUMO levels of the carbon cage. The changes switch the reactivity in the photolysis of **1**.

## 3. Cage-Opening Reactions of EMFs

**Figure 2:**
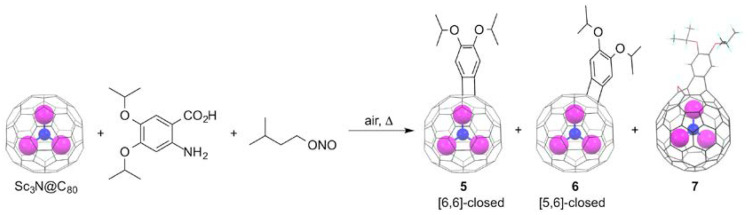
Cycloaddition reaction of Sc_3_N@C_80_ with 2-amino-4,5-diisopropoxybenzoic acid and isoamyl nitrite, to produce open-cage metallofullerene **7**.

Among the various chemical functionalizations of fullerenes, cage-opening reactions are those which enable one to gain access to the inner space of the otherwise “locked” carbon sphere. Wudl and co-workers first introduced the concept that creates an orifice on the carbon cage in 1995 [26]. Since then, several examples of open-cage fullerenes prepared from C_60_ and C_70_ have been developed. A prominent result is the fact that the open-cage fullerenes that enable the encapsulation of small molecules such as H_2_ [27,28], H_2_O [29,30], HF [31,32], and even CH_4_ [33] are available. Further orifice-closing reactions engender the creation of endohedral fullerenes. In contrast, cage-opening reactions of EMFs have remained unexplored until recently, due to the limited availability of EMFs and different chemical reactivities of EMFs from those of empty fullerenes. In 2011, Wang and coworkers found, by accident, that benzyne addition to Sc_3_N@*I_h_*(7)-C_80_ (hereinafter abbreviated as Sc_3_N@C_80_) yielded an oxygen-bridged open-cage EMF derivative **7** in addition to the [6,6]-benzyne adduct **5** and [5,6]-benzyne adduct **6**, when the reaction was conducted under air, as shown in Figure 2 [34]. Both **5** and **6** possess closed four-membered rings at the site of addition, which are formed through formal [2 + 2] cycloaddition of the in-situ generated benzyne to Sc_3_N@C_80_. The corresponding [4 + 2] cycloaddition does not occur because fullerenes do not act as dienes. The formation of **7** became dominant, when the reaction of Sc_3_N@C_80_ with 2-amino-4,5-diisopropoxybenzoic acid and isoamyl nitrite (reactant ratio: 1:5:8) was conducted in 1,2-dichlorobenzene (1,2-DCB) in the presence of 25 equiv. of water under aerobic conditions at 60°C for 12 h. Under these conditions, **5**, **6**, and **7** were obtained, respectively, in 7%, 3%, and 12% yields, in addition to the recovery of the pristine EMF in 46% yield. The single-crystal XRD studies of **7** revealed its unique molecular structure. First, the [5,6] C–C bond at the site of the benzyne addition is broken. Secondly, an oxygen atom was added to one of the [5,6] C–C bonds of the benzyne-attached pentagonal ring, in which the C–C bond is also broken to form not an epoxy, but an ether substructure on the cage. Consequently, the carbon cage of **7** contains a 13-membered ring orifice, although the orifice is spanned by an oxygen and a benzyne moiety. Presumably, **7** was formed by oxygenation of **6**, although details of the reaction mechanism for the formation of **7** remain unclear. In fact, both **5** and **6** were stable in air at 60°C for 12 h and at 180°C for 8 h.

**Figure 3:**
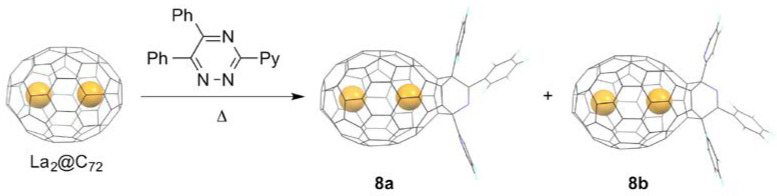
Cage-opening reaction of La_2_@C_72_ with 5,6-diphenyl-3-(2-pyridyl)-1,2,4-triazine.

To access the inner space of EMFs, no bridging moiety at the orifice is necessary. In this regard, an open-cage EMF bearing three seven-membered ring orifices without bridges was first reported in 2015 using La_2_@*D*_2_(10611)-C_72_ as the starting material, as shown in Figure 3 [35]. The dimetallofullerene La_2_@*D*_2_(10611)-C_72_, known as a non-IPR EMF, contains two pentalene units at opposite ends of the rugby ball-shaped carbon cage [36]. In this context, exploration for the chemical functionalization of La_2_@*D*_2_(10611)-C_72_ (hereinafter, the molecule will be abbreviated as La_2_@C_72_) is also important for elucidating the chemical reactivity of non-IPR EMFs. As a cage-opening, the thermal reaction of La_2_@C_72_ with 5,6-diphenyl-3-(2-pyridyl)-1,2,4-triazine at 180°C in a sealed tube was conducted. After 72 h, 68% of La_2_@C_72_ was consumed, and it was converted quantitatively to two bisfulleroid isomers (labelled as **8a** and **8b**) in a 1:1 ratio. Between them, the single-crystal XRD analysis of **8a** revealed its open-cage structure. The results suggest that the initial [4 + 2] cycloaddition took place at the [5,5] C–C bond, at one of the two pentalene units. After N_2_ extrusion, the subsequent electrocyclization and the following cycloreversion sequence produced the final open-cage structure. In this respect, the selective reaction at the [5,5] junction resulted from the highest reactivity of the central C–C bond of the pentalene units. However, the unique site selectivity of La_2_@C_72_ is not predictable based on the frontier orbital criteria. In fact, DFT calculations suggest that HOMO, LUMO+1, and LUMO+2 of La_2_@C_72_ are strongly delocalized over the whole sphere, whereas the LUMO is located mainly on the internal La atoms. Instead, the POAV values can be useful to explain the highest reactivity of the [5,5]-bond. The crystallographic determination of the molecular structure of **8b** is missing. However, it can be deduced that the first reaction step occurred at the same [5,5]-bond because the other diastereomer can be formed by the addition of the triazine to the bond in its reversed direction.

## 4. Selective Bis-Addition Reactions of EMFs

**Figure 4:**
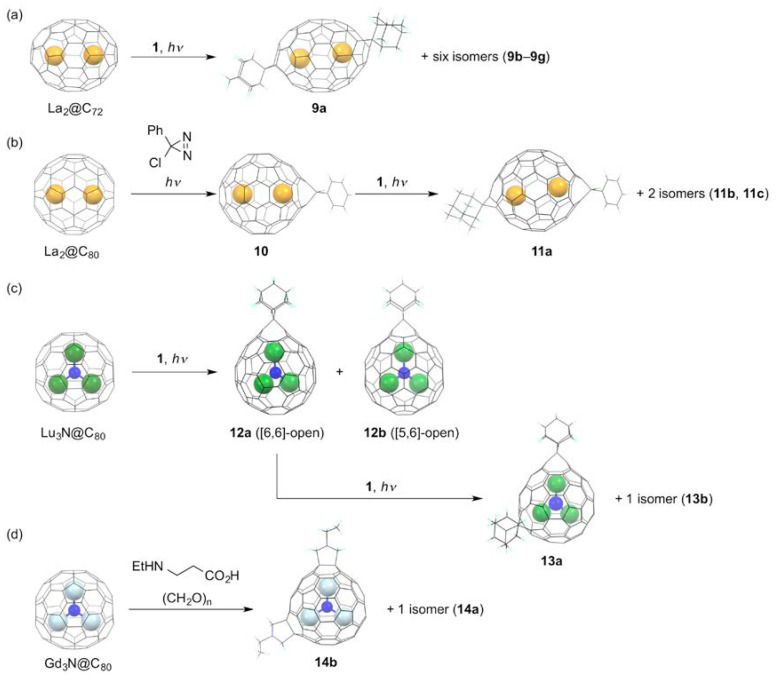
Bisfunctionalization of EMFs. (**a**) Photochemical reaction of La_2_@C_72_ and **1**. (**b**) Two-step bisfunctionalization of La_2_@C_80_. (**c**) Two-step bisfunctionalization of Lu_3_N@C_80_. (**d**) Prato reaction of Gd_3_N@C_80_ with *N*-ethylglycine and paraformaldehyde.

It is reasonable to consider that the site selectivity of EMFs is influenced by the internal metal species, because the charge density distribution of the cage carbons and the frontier MO distribution of the whole molecule are changed drastically by metal doping [8]. For instance, in La@*C*_2*v*_(9)-C_82_, the La atom is localized near the hexagonal ring along the *C*_2_ axis of the *C*_2*v*_ carbon cage. Consequently, the carbons close to the La atom are negatively charged, while, on the contrary, the carbons distant from the La atom are positively charged. As a result, electrophilic reactions tend to occur at the cage carbons close to the La atoms, whereas nucleophilic reactions tend to occur at the cage carbons at the opposite side. In this regard, dimetallic EMFs are likely to be suitable reactants for selective bis-addition reactions. The first example of the bisadduct formation of dimetallic EMFs was the photochemical reaction of La_2_@C_72_ with **1** reported by our group in 2008 [37,38]. In this reaction, seven isomers of La_2_@C_72_(Ad)_2_ (**9a**–**9g**) were isolated, of which the structure of the most abundant isomer **9a** was found using single-crystal XRD, as shown in Figure 4a. Results indicated that two Ad groups were added to the two pentalene regions at both poles of La_2_@C_72_, which face the encaged La atoms. Based on the high reactivity of the pentalene regions, the two Ad groups are likely to have added to the two poles in the other bisadduct isomers.

In contrast to the rugby-ball-shaped La_2_@ C_72_ molecule, the selective bis-addition reaction of La_2_@*I_h_*(7)-C_80_ was not straightforward, due to its round structure and three-dimensional dynamic motion of the encaged La atoms [39]. At this point, a stepwise addition reaction protocol is an alternative pathway to obtain the corresponding bisadducts regioselectively [40]. As the first step, the photochemical reaction of La_2_@*I_h_*(7)-C_80_ with phenylchlorodiazirine was conducted, as shown in Figure 4b. The resulting monoadduct, [6,6]-open La_2_@*I_h_*(7)-C_80_(CClPh) (**10**), features the two encaged La atoms collinear with the spiro carbon of the attached Ad moiety [41]. Regulation of the motion of the La atoms is attributable to the C–C bond breaking of the addition site, which engenders elongation of the La···La distance. As a result, the two La atoms are localized respectively at the position close to the addition site and the opposite side of the addition site. Therefore, the latter site is expected to be more reactive toward an electrophile. As the second step, the photochemical reaction of **10** with **1** proceeded smoothly to afford three isomers of the bisadduct La_2_@*I_h_*(7)-C_80_(CClPh)(Ad) (**11a**–**11c**). One can notice that the second carbene addition afforded only three isomeric adducts, although many possible addition sites exist in **10**. Single-crystal XRD studies of **11a** have revealed that the two addends are attached to both ends of the La_2_@*I_h_*(7)-C_80_ molecule. The Ad addition coincided with the C–C bond cleavage at the site of addition. These studies indicate clearly that the encaged metal atoms in EMFs can regulate the reactive sites.

This is also in the case for trimetallic nitride-templated EMFs (TNT-EMFs). In early works, Gibson, Dorn, and coworkers reported that the Bingel–Hirsch reaction of Sc_3_N@*D*_3*h*_(5)-C_78_ with diethyl bromomalonate in the presence of DBU afforded a highly symmetrical bisadduct Sc_3_N@*D*_3*h*_(5)-C_78_[C(CO_2_Et)_2_]_2_, in addition to the monoadduct Sc_3_N@*D*_3*h*_(5)-C_78_[C(CO_2_Et)_2_] in 2008, although the addition site was not fully characterized [42]. In 2017, our group reported the first example for the X-ray crystallographic structure of a TNT-EMF bisadduct, as shown in Figure 4c [43]. In Lu_3_N@*I_h_*(7)-C_80_, the encaged metal cluster exhibits three-dimensional random motion in the carbon sphere. Consequently, a stepwise strategy was adopted for regioselective bisfunctionalization. As the first step, the photochemical reaction of Lu_3_N@*I_h_*(7)-C_80_ with **1** afforded [6,6]-open Lu_3_N@*I_h_*(7)-C_80_(Ad) (**12a**) as the major product, and [5,6]open Lu_3_N@*I_h_*(7)-C_80_(Ad) (**12b**) as the minor product. The X-ray crystallographic structures of **12a** and **12b** disclosed that one metal site was positioned near the addition site. The other two Lu atoms were disordered and positioned at eight sites. The results indicated that the Lu_3_N cluster is allowed to rotate similarly to a spinning top inside the carbon sphere. The subsequent photochemical reaction of the **12a** with **1** proceeded regioselectively to afford two bisadducts (**13a** and **13b**) that were isolated by subsequent HPLC separation. The X-ray structure of **13a** showed that the second Ad group was attached at a [6,6] bond near a Lu atom in a [6,6]-open fashion. Therefore, one can reasonably state that the regioselectivity in the Ad bisaddition is regulated by the triangular structure of the endohedral Lu_3_N cluster. Actually, DFT calculations suggest that the X-ray determined bisadduct isomer is the most stable one among five candidates.

Later, Yamakoshi et al. reported the X-ray structure of a bis-ethylpyrrolidinoadduct of Gd_3_N@*I_h_*(7)-C_80_ (hereinafter, the molecule is abbreviated as Gd_3_N@C_80_) prepared by 1,3-dipolar cycloadditions (so-called Prato reaction [44,45,46,47,48,49]) of Gd_3_N@*I_h_*(7)-C_80_, with excess amounts of *N*-ethylglycine and paraformaldehyde in 2019 [50]. The reaction yielded two isomers of the bisadducts (**14a** and **14b**), in addition to the corresponding monoadduct, as shown in Figure 4d [51]. The crystal structure of the minor isomer **14b** exhibited a *C*_2_-symmetric [6,6][6,6]-structure. The addition sites of the minor isomer of Gd_3_N@*I_h_*(7)-C_80_[(CH_2_)_2_NEt]_2_ differ from those of **13a**, even though two of the three Gd atoms of the internal Gd_3_N cluster are positioned close to the sites of the additions, as expected. The Gd_3_N cluster was flattened out in the crystal structure of the minor bisadduct, as compared with that in pristine Gd_3_N@*I_h_*(7)-C_80_. The planarization can be attributed to the release of the strain energy of the cluster by filling out the increased locally available endohedral space in the bisadduct. A portion of **14b** isomerized to **14a** under a thermal condition, although isomerization of another isomer as well as retrocycloaddition also proceeded simultaneously. The results indicated **14b** as a kinetic product, whereas the major bisadduct was a thermodynamic product. X-ray crystallographic identification of **14a** has been lacking. However, the authors proposed that **14a** also possesses a [6,6][6,6]-structure, although several candidates have similar relative stabilities. They also proposed that the isomerization can proceed via a “walk on the sphere” rearrangement, as observed in bis-malonate adducts of C_60_ under electrochemical conditions [52]. It is noteworthy that the two addition sites in the proposed structure of **14a** are the same as those in **13a**. At present, only the major products have been characterized while characterization of other minor products has been lacking. Future efforts will be devoted to the characterization of minor products to compare the regioselectivity in Bingel vs. 1,3-dipolar cycloadducts for EMFs and empty fullerenes.

## 5. Single-Bonding Derivatization of EMFs

When an odd number of electrons are formally transferred from the internal metal species to the carbon cages, the EMFs possess an open-shell electronic state. Such open-shell EMFs are stabilized by the delocalization of the unpaired electron over the molecule. The chemical reactivity of open-shell EMFs has also been explored [8]. Results have revealed that several reactions of open-shell EMFs, particularly those involving radical species, afforded singly bonded derivatives bearing a closed-shell electronic state [53,54,55,56]. Such single-bonding derivatization ability yielding closed-shell products is apparently a general trend for open-shell EMFs, such as trivalent M@C_82_. In related work, we found that an open-shell EMF, Ce@*C*_2*v*_(9)-C_82_, was dimerized in its cocrystals with nickel octaethylporphyrin [57]. Similar dimerization was also apparent in the cocrystals of Y@*C_s_*(6)-C_82_ [58] and Er@*C_s_*(6)-C_82_ [59] with nickel octaethylporphyrin. In addition, functionalized EMFs bearing open-shell electronic species, such as La@*C*_2*v*_(9)-C_82_[CH(CO_2_Et)_2_]_2_ [60] and La@*C*_2*v*_(9)-C_82_(Cp*) (Cp* = 1,2,3,4,5-pentamethylcyclopentadiene) [61], were dimerized in their crystals. In stark contrast, the monoaddition of a radical to closed-shell EMFs can yield fullerenyl radicals. In fact, the corresponding C_60_-based fullerenyl radical R-C_60_• can be generated by nucleophilic addition and subsequent one-electron oxidation, or by thermal dissociation of single-bonded fullerene dimers [62]. However, fullerenyl radicals are usually very reactive. They cannot be isolated because of their high radical reactivity. The results of the EPR studies suggest that an unpaired electron was not delocalized over the carbon sphere, but rather confined to the carbon at ortho and the two carbon atoms para to the sp^3^-hybridized cage carbon bound with R in R-C_60_•.

**Figure 5:**
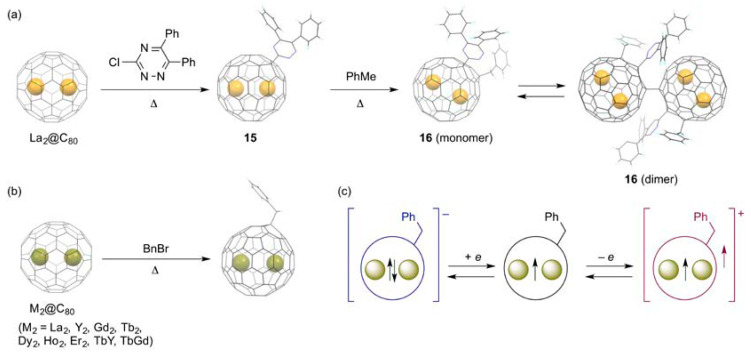
(**a**) Reaction of La_2_@C_80_ with 3-chloro-5,6-diphenyltriazine, and reaction of **15** with toluene. (**b**) Benzylation of M_2_@C_80_. (**c**) Schematic description of the single-electron reduction and oxidation of M_2_@C_80_(CH_2_Ph).

By contrast, we discovered, in 2015, that the monoaddition of a radical to a closed-shell EMF, La_2_@*I_h_*(7)-C_80_, afforded a singly bonded fullerenyl radical as an air-stable product, as shown in Figure 5a [63]. The fullerenyl radical, La_2_@*I_h_*(7)-C_80_–C_3_N_3_Ph_2_ (**15**), was synthesized by the thermal reaction of La_2_@*I_h_*(7)-C_80_ and excess amount of 3-chloro-5,6-diphenyltriazine, under reflux in 1,2-DCB. The product was readily isolated by preparative HPLC. Subsequently, XRD analysis showed that the addition of the carbon-centered triazinyl radical took place at one of the carbon atoms shared by one pentagon and two hexagon rings, i.e., [5,6,6]-carbon atoms. The absorption spectrum of **15** exhibits broad absorption bands over the near-IR region down to 1300 nm, as a consequence of the paramagnetic nature. At this point, no meaningful NMR spectra were observed in **15**. The paramagnetic character was confirmed from X-band EPR measurements, showing that the EPR spectrum of **15** resembled that of La_2_@*I_h_*(7)-C_80_ anion radical. Detailed analysis revealed that a large spin density is associated with each La atom, and that each La atom has the same spin density. In fact, the LUMO of La_2_@*I_h_*(7)-C_80_ is well-known to be associated with the La–La σ-bonding orbital [64,65]. In this context, it is reasonable to state that the unusual stability of the fullerenyl radical in air arises from the confinement of the unpaired electron to an internal, metal–metal bonding orbital. The short La–La distance of 3.78 Å found in the crystal structure of the fullerenyl radical is consistent with this consideration. The chemical reactivity of the fullerenyl radical was also investigated. In fact, heating of the mixture of **15** and an excess amount of toluene in 1,2-DCB afforded addition of a benzyl to yield a closed-shell product, La_2_@*I_h_*(7)-C_80_–(C_3_N_3_Ph_2_)(CH_2_Ph) (**16**), in a 1,4-addition form. The product shows no EPR signals in solution due to the diamagnetic character. In addition, the longest absorption band was red-shifted in the absorption spectrum of **16** in solution, when compared with that of **15**. It is particularly interesting that this closed-shell product formed the open-shell dimer in its crystal. In this dimer, each unpaired electron is again confined to an internal La–La bonding orbital of each La_2_@*I_h_*(7)-C_80_ unit, to be a diradical. In fact, a broad signal was observed in the EPR spectrum of the crystal.

Triggered by this success, several groups have prepared and characterized similar fullerenyl radical derivatives of M_2_@*I_h_*(7)-C_80_ (hereinafter, the molecules are abbreviated as M_2_@C_80_). Lu et al. synthesized a benzyl derivative of La_2_@C_80_ by the photochemical reaction of La_2_@C_80_ with benzyl bromide in toluene; they have reported the short La–La distance (3.68–3.78 Å) in its crystal, as found in the crystal of **15** [66]. Later, Popov et al. also synthesized similar air-stable benzyl monoadducts of Y_2_@ C_80_ and Dy_2_@C_80_ by the reaction of the fullerene mixtures containing M_2_@C_80_ (M = Y, Dy) in DMF with benzyl bromide in 2017, as shown in Figure 5b [67]. They showed that giant exchange interactions between lanthanide ions and the unpaired electron caused single-molecule magnetism of Dy_2_@C_80_(CH_2_Ph), with a record-high 100 s blocking temperature of 18 K, and a high thermal barrier of magnetization reversal of 613 K. Subsequent surveys of an array of M_2_@C_80_(CH_2_Ph) (M_2_ = Y_2_, Gd_2_, Tb_2_, Dy_2_, Ho_2_, Er_2_, TbY, TbGd) revealed that the strong ferromagnetic coupling emerging from the delocalization of the unpaired electron spin, which glues the magnetic moments of the two lanthanide ions together, is responsible for the high-spin magnetic ground states in the singly bonded derivatives [68]. Particularly, Tb_2_@C_80_(CH_2_Ph) showed giant coercivity and the highest known blocking temperature among dinuclear lanthanide complexes [69,70]. Additionally, they showed singly bonded derivatives. These derivatives are redox-active. Moreover, reversible one-electron oxidation and one-electron reduction were observed in their cyclic voltammograms, indicating that the single-molecule magnetism can be tuned electrochemically, as shown in Figure 5c.

**Figure 6:**
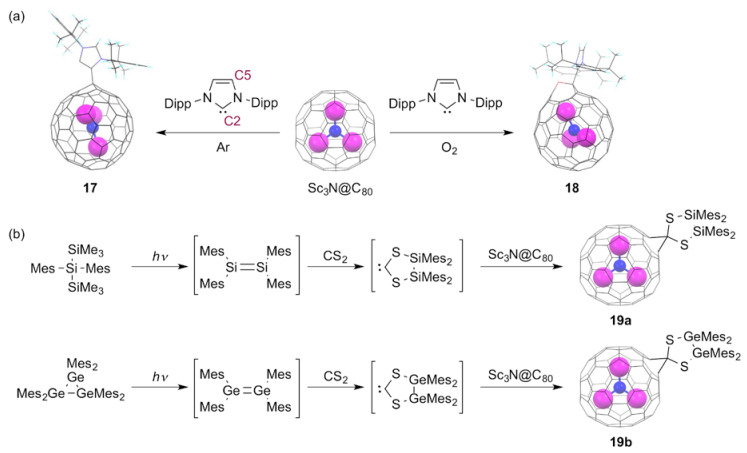
(**a**) Reactions of Sc_3_N@C_80_ with 1,3-bis(diisopropylphenyl)-imidazol-2-ylene in the absence or presence of oxygen. (**b**) Reactions of Sc_3_N@C_80_ with in situ generated SHCs.

In 2011, Bazan and coworkers demonstrated that empty fullerenes such as C_60_ and C_70_ can behave as all carbon π-Lewis acids when combined with *N*-heterocyclic carbene (NHC) as counterparts [71]. Their results showed that a singly bonded C_60_ derivative bearing a strong zwitterionic character was obtained as a Lewis acid–base adduct. In this context, the chemical reactivity of EMFs toward NHCs has been attracting growing interest to elucidate the π-Lewis acidity of the negatively charged EMF carbon cages [72]. Lu et al. reported the that reaction of Sc_3_N@C_80_ with 1,3-bis(diisopropylphenyl)-imidazol-2-ylene (IDipp) afforded a singly bonded derivative, [6,6,6]-Sc_3_N@*I_h_*(7)-C_80_-IDipp (**17**) [73]. In addition, the XRD analysis of **17** demonstrated that the NHC bonded to the Sc_3_N@C_80_ cage with its “abnormal” carbene center C5, instead of the normal site C2, which differs from the corresponding C_60_–IDipp adduct, as shown in Figure 6a. Subsequent DFT calculations suggested that the formation of the [6,6,6]-adduct was not a thermodynamically controlled process. It is particularly interesting that introducing a small amount of oxygen in the reaction mixture induces formation of different products. In fact, the reaction of M_3_N@C_80_ (M = Sc, Lu) with IDipp in the presence of oxygen afforded not only the C5-linked [6,6,6]-M_3_N@C_80_-IDipp, but also the oxidized derivative of C2-linked [6,6,6]-M_3_N@C_80_-IDipp (**18**) [74]. It is proposed that the addition of oxygen can release the steric hindrance between the bulky NHC moiety and the EMF cage.

The reactivity of NHCs toward EMFs depends on the bulkiness of the substituents of NHC as well as on the cage structure and the encapsulated species of EMFs used. For instance, the reaction of Sc_2_C_2_@*C*_3*v*_(8)-C_82_ with IDipp in the absence of oxygen proceeded regioselectively, to afford two products [75]. Subsequent XRD analysis of one product showed that the product is a singly bonded derivative, [5,6,6]-Sc_2_C_2_@*C*_3*v*_(8)-C_82_-IDipp, in which IDipp is linked to the EMF cage with its normal carbon center C2. In contrast, the reaction of Lu_2_@C_82_ species (C_82_ = *C*_3*v*_(8)-C_82_, *C*_2*v*_(9)-C_82_) with 3-dimesityl-1*H*-imidazol-3-ium-2-ide (IMes) in the presence of oxygen afforded C2-linked [5,6,6]-Lu_2_@C_82_-IMes as a single product without formation of any oxidized product [76]. These results suggest that the high regioselectivity and preferential formation for monoadducts are determined mainly by the electronic effect of the EMF carbon cages and the steric repulsion between the NHC and EMFs. In fact, DFT calculations suggest that the coverage of the cage carbons featuring both large LUMO distribution and positive electrostatic potential values by the bulky NHC group can engender prevention of further additions. In contrast to the reactivity of NHCs, the reactions of *S*-heterocyclic carbenes (SHCs), generated in situ by the cycloaddition of disilenes and digermenes to CS_2_ with Sc_3_N@*I_h_*(7)-C_80_, afforded the corresponding methano-bridged derivatives (**19a,b**), as shown in Figure 6b **[77]**. Although the XRD characterization is lacking, NMR spectral analyses suggest that these derivatives possess the corresponding [6,6]-open structures.

## 6. Summary

This review has highlighted the unique chemical reactivity of EMFs, which differs from that of empty fullerenes. The structural diversity of EMFs has provided extreme variation in chemical reactivities. The resulting derivatives exhibited interesting properties derived from the combination of the cage structures, encaged metal species, and the functional groups bonded to the carbon cages. For instance, recent results have demonstrated clearly that the site of addition in the carbon cage is governed by metal-atom doping. This is also the case for the second addition. Particularly, the location of the internal metal atoms strongly affects the site-selectivity of EMFs. Selective bisfunctionalization of EMFs is expected to be valuable for the construction of EMF-based functional materials. In this respect, further efforts are anticipated to address selective trisfunctionalization using the triangular structures of trimetallic nitride clusters. Cage opening of EMFs to modulate the internal, untouchable metal species has remained challenging. However, recent studies of this aspect have led to remarkable progress in the first step of the molecular surgery of EMFs. The discovery of air-stable EMF-based fullerenyl radicals featuring confinement of an unpaired electron on the internal orbital paves the way to EMF-based single molecular magnets bearing giant exchange interactions. In addition, exploring the use of the π-Lewis acidity of EMF carbon cages by combining with heterocyclic carbenes provides novel zwitterionic EMF derivatives. Studies of EMFs will continue to gain importance in achieving the construction of multifunctional molecules for applications.
